# Co-occurring Mental Disorders in Transitional Aged Youth With Substance Use Disorders – A Narrative Review

**DOI:** 10.3389/fpsyt.2022.827658

**Published:** 2022-02-24

**Authors:** Patrick Köck, Maximilian Meyer, Julie Elsner, Kenneth M. Dürsteler, Marc Vogel, Marc Walter

**Affiliations:** ^1^Department of Addictive Disorders, University Psychiatric Clinics Basel, Basel, Switzerland; ^2^University Psychiatric Clinics Basel, Clinic for Children and Adolescents, University of Basel, Basel, Switzerland; ^3^Department for Psychiatry, Psychotherapy and Psychosomatic, Psychiatric Hospital, University of Zurich, Zurich, Switzerland; ^4^Division of Substance Use Disorders, Psychiatric Clinic, Psychiatric Services of Thurgovia, Münsterlingen, Switzerland; ^5^Department of Psychiatry and Psychotherapy, Psychiatric Services Aargau, Windisch, Switzerland

**Keywords:** adolescence, addiction, mental health, illicit substance use, young adults, personality disorders, ADHD, psychosis

## Abstract

Adolescence and emerging adulthood are often referred to as youth. Transitional psychiatry addresses this target group, which considers patients between 15 and 25 years of age. Substance use usually begins and peaks at this stage of life. Psychiatric disorders, foremost attention-deficit/hyperactivity disorder, and affective disorders, conduct disorders, and first-episodes psychosis frequently appear in early life stages. This review aims to provide a broad overview of transitional-aged youth's most common psychiatric comorbidities with substance use disorders. A literature search was conducted in Embase and Pubmed, and the main findings are described narratively. We present main findings for the following comorbidities: attention-deficit/hyperactivity disorder, conduct disorder, personality disorders, affective disorders, psychotic disorders, and the phenomena of overdose and suicidality. In conclusion, co-occurring mental health disorders are common and appear to facilitate the development of substance use disorders and exacerbate their overall course. Substance use also affects the severity and course of comorbid psychiatric disorders. Overall, data on transition-age youth with substance use disorders are highly inconsistent. Universal screening and treatment guidelines do not yet exist but should be aimed for in the future.

## Introduction

Adolescence is a crucial phase of life in many respects. It is characterized by adolescent autonomy strivings, exploration behavior and peer orientation. Scholars have proposed the developmental transformation in the adolescent brain to be particularly vulnerable regarding sensitivity to neurotoxicity and the development of substance use disorders (SUD) (1, 2). This is relevant, as initiation of drug use peaks in adolescence but overall drug use followingly decreases after this period (3). Therefore, most adolescents do not show problematic use patterns and this spike can be understood as experimental drug use (4). Nonetheless, the highest risk for onset of use and development of addiction occurs in early adolescence until ~ age 25 (5, 6).

Transitional psychiatry specializes in treating the population transitioning from childhood to adulthood (transitional aged youth, TAY). Although a uniform definition of the age range has not been established, TAY usually addresses adolescents between 16 and 24 (7) to 26 years (8). Another term for this population found in literature is “emerging adulthood”, with the proposed definition of 18–25 years of age (9). Global prevalence rates for SUD in adolescents and emerging adults are difficult to obtain. The prevalence rates vary highly due to several reasons, such as availability of data, geographic factors, cultural differences, and the varying definitions of TAY. Global or European large scale data collection projects concerning substance use, i.e., the World Drug Report (10), the European Drug Report (11) and the European School Survey Project on Alcohol and Other Drugs (12), either focus on availability of drugs or use prevalence without differentiating by age group. Other challenges in gathering data on problematic substance use or SUD in TAY include early treatment terminations and that many adolescents do not seek or receive treatment (13, 14).

Co-occurring mental disorders in individuals with SUD or “dual diagnoses” pose a particular clinical challenge in the treatment of SUD (15). Mental disorders can negatively influence or exacerbate SUD and often lead to unsatisfactory treatment outcomes or undertreatment of co-occurring disorders (16). Hence, dual diagnoses necessitate special clinical consideration during the treatment of these patients and often require the integration of multiple therapeutic strategies (17), such as multisystemic therapy for example (18). As with prevalence rates, literature on dual diagnoses in the TAY population is scarce. However, epidemiological data shows that the age of onset for 62.5% of mental disorders is 25 years or below (19). Earlier US studies estimate the median prevalence of function-impairing child and adolescent psychiatric disorders at 12% (20). It has also been found that preexisting mental disorders represent risk factors for the emergence and progression of SUD (21). A study of juvenile detainees demonstrated that substance use, behavioral disorders, and internalizing disorders predict the development of SUD. This underlines the importance of treating childhood/adolescent disorders to reduce the emergence of secondary SUDs (22). These findings further indicate that individuals diagnosed with a mental disorder relatively often have more than one diagnosis.

In this narrative review, we screened the literature until November 2021 using Embase and Pubmed. The search was conducted in September and October 2021 by our study group. All literature until October 31, 2021, was considered for this review. The following terms were used in the search strategy: “substance use disorder”, “comorbidity”, “juvenile”, “adolescence”, “youth”, “transitional psychiatry”. Only peer-reviewed articles listed in either Embase or Pubmed reporting on human data were considered for this review. Substance use disorders, in the context of this study, include disorders related to illicit substances and legal addictive substances such as nicotine, alcohol, or prescription medications.

Based on clinical experience with patients aged 15 to 25 years and unpublished data from our study group, we decided to weight the focus of this review accordingly. In the daily practice in a specialized outpatient clinic for TAY with SUDs (Ambulanz für Suchttherapie, AfS, University Psychiatric Clinics Basel), we encounter affective disorders, attention deficit/hyperactivity disorder (ADHD), and personality disorders as the most frequently diagnosed comorbidities. Substance use associated with psychotic disorders, suicidal crisis and/or overdoses pose major challenges for clinicians. Furthermore, conduct disorder often co-occur with SUD and ADHD, and often precedes personality disorders. Hence, we have narrowed our study selection to ADHD, conduct disorder, personality disorders, affective disorders, psychotic disorders, suicidality and overdose phenomena. This review is not conclusive but should rather provide a broad overview of this relevant yet complex topic. Therapeutic recommendations are beyond the scope of this narrative review.

## Co-Occurring Mental Disorders and Phenomena of Substance Use Disorders in Youth

### Attention-Deficit/Hyperactivity Disorder

The link between childhood attention-deficit/hyperactivity disorder (ADHD) and early SUD in youth and young adults has been well-established (23, 24). Skoglund et al. (25) found that not only the affected but also relatives of individuals with ADHD are more likely to develop SUDs (25). Despite the lack of information about the prevalence of co-occurring ADHD and SUD in the general population, studies have shown that at least one fourth of adolescents suffering from SUD also suffer from ADHD (26–29). This can partially be explained by either general vulnerability, genetic predispositions, or dysfunctions in the inhibitory and reward related neural circuits of the brain (30, 31). Consequently, symptoms of ADHD such as inattention, hyperactivity, and impulsivity may increase behavioral and social problems, as well as risk and drug seeking behavior (23, 32). Conversely, it seems less likely that substance use leads to ADHD since the age of onset of the latter generally predates first drug or alcohol use (33, 34).

Data is inconsistent regarding the risk of developing SUD in ADHD. Some studies found no significant effect of ADHD on SUD itself but rather that the connection between ADHD and SUD was almost entirely linked to conduct disorder (CD) (35–37). However, other studies reported on higher nicotine, alcohol, and illicit drug use in ADHD patients independent of CD (32). A meta-analysis by Özgen et al. found that the risk of SUD was highest in children with concurrent ADHD and CD (23, 38). Katusic et al. reported that individuals with comorbid ADHD and CD were 8.8 times more likely to have any SUD before the age of 18 years compared to those with ADHD alone (39). Besides, patients with ADHD were more prone to develop SUDs earlier and more severely (29, 40). Importantly, over 70% of TAY with SUD and ADHD also suffer from oppositional defiant disorder (ODD), CD, or both (41). Other predisposing factors for SUD co-occurrent with ADHD were bipolar disorder, eating disorder, low socioeconomic status, and dropping out of high school.

Across the literature, ADHD is more common in males with rates ranging between 3:1 and 1.5:1 in population-based studies (23). Also, ADHD seems to be detected and diagnosed less often in females (42, 43) which in turn can lead to delayed and insufficient treatment and more severe outcomes over time in female patients (23). Gender differences have also been observed regarding the age of onset, type of symptoms, use of medication, and quality of life (44). Regarding the prevalence of comorbid ADHD in SUD patients, one study found no sex differences (26). However, other studies indicate that females with ADHD are slightly more likely to have comorbid SUDs than males with ADHD (44). An adolescent twin study conducted by Disney et al. also suggests that girls tend to be more vulnerable to develop SUDs (37).

Studies of drug- and alcohol-dependent populations have brought up self-medication hypotheses in the context of aggressive, anxious, or hyperactive symptoms as possible explanations for the development of SUDs (45, 46). For instance, a longitudinal study by Kollins et al. showed that patients with ADHD are at increased risk of initiating smoking and nicotine dependence (47). In the past, data regarding stimulant use in patients with ADHD and SUD have been heterogenous (48). However, some investigators found that males treated with psychostimulants were less likely to develop SUDs than those who did not receive stimulant treatment (49). Yet, no such protective effects were described in girls (39). Wilens et al. found an overall reduced risk of developing any SUD in later life (mean *OR* = 1.9) in patients who had received stimulant treatment than those who had not (50). In line with these findings, reviews suggest that stimulant treatment of childhood ADHD may have a preventive effect on the development of SUD in adolescence and early adulthood (23). More specifically, Groenman et al. described that stimulant treatment at an early stage, high dose, and longer duration were associated with a reduced risk of SUD in adolescence (51, 52). Other authors concluded that treatment initiated during elementary school might reduce the risk of developing any SUD (excluding nicotine) over time. In contrast, initiation of psychostimulant treatment during adolescence may heighten SUD risk in certain subgroups (53).

### Conduct Disorder

Conduct disorder (CD) usually develops in childhood or adolescence and affects around 3% of schoolaged children. CD is more prevalent in males than in females (2:1) and is characterized by heterogenous patterns of aggression and antisocial behavior. Symptom profiles of CD are complex and highly heterogenous (54). Current diagnostic criteria in the Diagnostic and Statistical Manual of Mental Disorders (DSM-IV and DSM-V) require a minimum of three out of 15 symptoms for the diagnosis of CD. Hence, 32,647 symptoms patterns could lead to the diagnosis of CD (55). Due to its heterogeneity and complexity CD could be positioned somewhere between or alongside ADHD and personality disorders mainly for two reasons. Firstly, co-occurrence with ADHD is frequent and symptoms can overlap. Secondly, CD can often lead to the development of antisocial personality disorder (ASPD) later in adulthood (56). Additionally, another study group also found ADHD, with or without comorbid CD, to increase the risk for the later development ASPD (57). CD in adolescence is associated with a heightened risk of initiating substance use (58). Although early-onset drug use is associated with later ASPD, conduct disorder may precede and predict substance use (37) and which may be a Pre-requisite for the development of some forms of chronic ASPD. Though the interaction between substance use and CD is not yet completely understood, CD displays a severe burden. A study from the US of youth with CD and co-occurring SUD showed that these patients and their siblings display a heightened risk of premature death compared to similar controls (59).

### Personality Disorders

The relationship between SUD and co-occurring personality disorders (PD) in adults is well-established (60–63). A recent, larger-scaled epidemiological US study showed significant associations between the prevalence of any SUD with borderline, antisocial, and schizotypal PD (64). These findings for adults have been widely accepted across the literature (65). However, data regarding PDs in TAY with problematic substance use are limited (66).

An earlier attempt of analyzing the association between comorbid PD and SUD in hospitalized adolescents was made by Grilo et al. (67). They found an overall elevated rate of PD in patients with SUD compared to those without SUD. The authors attribute their findings to the assumption that substance use is partly elicited due to impaired impulse control (67). In a more extensive, prospective analysis of detained youth (12–17 years), generalized anxiety disorder, ADHD, and conduct disorders predicted the later onset of SUD. Substance use and behavioral disorders affected one in six males, in a longitudinal study of 1829 TAY patients after detention. This was the most common comorbidity profile among all participants (22). CD and early onset of alcohol or drug use are also associated with the later development of antisocial personality disorder. (ASPD) (22, 37).

A study of first-time admitted psychiatric inpatients with SUD (mean age = 31.4 years) showed that 46% suffered from comorbid axis II disorders and comorbid axis I disorders were found in 85%. Among the sample, only 7% displayed a comorbid disorder classified solely as “substance-induced”, whereas other comorbidities were considered independent or partly independent from substance use (68). An in-depth analysis of the same sample showed that the psychiatric symptom load [measured with the Symptom Check List (SCL-90), Inventory of Depressive Symptoms (IDS) and the Global Assessment of Functioning (GAF)] of patients with SUD and comorbid PD was more severe. Patients with PD were younger at time of onset and psychiatric hospitalization. Also, their research suggests the requirement of different treatment approaches and recommends early assessment of PD in patients with SUD. Interestingly, the authors found Cluster C disorders (“anxious” or “fearful” such as paranoid, schizotypal, avoidant or schizoid personality disorders) to be as prevalent as Cluster B disorders (“dramatic” or “overly emotional”, such as histrionic, borderline, narcissistic or antisocial personality disorders) (66).

In Korsgaard et al. (69) screened 153 adolescents that had been referred to an outpatient mental health clinic. 18.3% screened positive for SUDs. Whereas no gender difference in the prevalence of SUDs were observed, the association of PD and SUD was found significantly more often in females. These findings suggest that adolescent girls suffering from PD who present to treatment may be particularly vulnerable to developing SUDs (69). Preliminary results of another study of adolescents indicate that personality traits such as high neuroticism, low conscientiousness, and low agreeableness may contribute to the development and persistence of SUD as adolescents transition to adulthood (70). A longitudinal twin-study indicates that in adolescence, the co-occurrence of borderline personality disorder (BDP) traits and substance use effects from common risk factors rather than caused by one risk factor being a causal precursor of the other (71). Other researchers conducted a within-person analysis of the association between alcohol use and symptoms of BPD. Their results indicate aggravated BPD symptoms following an increased frequency of alcohol use in adolescent girls (72).

Another recent review, which was not exclusively focused on TAY, found that different attachment patterns are associated with the use of various substances. For example, fearful-avoidant attachment was frequent in heroin users, while more heterogeneous traits were found in patients with alcohol use disorder. However, in adolescence, insecure attachment patterns seemed more strongly associated with substance use than later in life (73) and fearful attachment was positively correlated with addiction severity scores in adolescents (74).

On the one hand, these findings display that certain personality traits or PD in adolescence predispose or heighten the vulnerability for developing a SUD. On the other hand, longitudinal data shows that early-onset regular tobacco, marijuana, and alcohol use is associated with the later manifestation of externalizing (such as ASPD) and internalizing mental disorders (75).

### Affective Disorders

Affective disorders, mainly depression, are among the most prevalent co-occurring diseases in TAY with SUDs (76). An early review reported that the estimates of concurrent depression in TAY with substance use ranged from 11.1 to 32.0%, with the mean being 18.8% (27). Conversely, in a Finnish sample, 17% of depressed TAY also suffered from SUDs (77). This suggests a strong link between SUDs and depressive disorders in this vulnerable age group.

Literature also indicates a link between SUDs and bipolar disorder in youth. In an Italian sample of 117 children and adolescents (aged between 7 and 18 years) with bipolar disorder, 27.4% also fit the criteria for SUDs (age *M* = 15.6; *SD* = 1.93) (78). This finding is consistent with earlier studies that found elevated prevalence rates of SUDs in youth with a history of mania and that the emergence of SUDs tended to Post-date the onset of bipolar disorder (79).

Data on depression in specific substance use disorders in TAY are fragmented. Several studies found higher rates of depression in TAY with amphetamine use. For example, the Victoria Adolescent Health Cohort Study found adolescents with depressive symptoms to be at over twice the risk of concurrent amphetamine use (80). In a US sample, 29.9% of 12–17-year-old methamphetamine users had suffered from major depression in the past year, whereas the prevalence for nonmedical ADHD medication users was 20.9% (81). Additionally, a significant association between symptom levels of depression and methamphetamine use was found in a Thai sample aged between 15 and 21 years (82).

Depression is among the most common co-occurring mental disorders in alcohol use disorder in youth. In a Greece sample of 16–18-year-olds, those with depression reported hard liquor use at least once a week in 24.5% (OR: 1.85; CI: 1.27–2.70) (83). The prevalence of depression in Non-using 9 to 18-year-olds increased from 5.0 to 23.8% in youth who used alcohol weekly (84). In a Finnish sample with a mean age of 17.5 years, early-onset depression predicted alcohol use frequency and recurrent intoxication (85). A study investigating 16 to 19-year-olds found that individuals suffering from alcohol use disorder were six times more likely to have a history of major depression (86).

As with adults, cannabis use in TAY is strongly linked to depression (87). A 10-year prospective study with a European sample of 14–17-year-old participants found that a history of major depression predicted cannabis use and cannabis use disorder onset, and lifetime prevalence for depression was higher in cannabis users when compared to Non-users (88).

### Psychotic Disorders

Epidemiological data, especially regarding psychosis and substance use in youth, is scarce. First-episodes of psychosis typically manifest between 15 and 30 years of age (89). First psychotic episodes commonly appear in late puberty or early adulthood (90). While the typical age of onset in females was observed between 15 and 25 years, a slightly earlier first onset was found in male patients (91, 92). Various illicit and prescribed substances can potentially cause psychotic states. Of all psychosis-inducing substances, cannabis and cannabinoids are the best-studied and most commonly used illicit drugs (93, 94). Also, stimulants like amphetamines, cocaine, methamphetamines, and methylphenidates are well-known for their psychotomimetic qualities. But also, nicotine, certain prescription medications, novel psychoactive substances, hallucinogens, and plant derivates (e.g., kratom) are associated with psychosis (95). Hence, differential diagnostics are of crucial importance in working with TAY. However, distinguishing between first episodes of primary psychotic disorders and substance-induced psychosis presents a major clinical challenge (95).

A Norwegian study of patients undergoing treatment for psychotic disorders found an annual incidence rate of substance-induced psychosis of 6.5/100 000. They further found incidence rates of 9.7/100 000 per year for psychosis patients with substance use vs. 24.1/100 000 in patients with psychosis without substance use (96). An Australian research group studied treatment-seeking youth and found that adolescents who are at-risk for psychosis [measured by The Positive Symptom Scale of the Comprehensive Assessment for At-Risk Mental State (CAARMS)] showed higher rates of nicotine, alcohol, and cannabis use (97). Other researchers report rates of SUD in patients with first-episode psychosis from 30 to 70% (98). These findings align with previous research, which showed that SUD are common in the early stages of psychotic disorders and individuals with first-episode psychosis (99, 100). In a sizeable Danish cohort study, diagnosis of schizophrenia was associated with subsequent development of SUD (101). Data from the same Danish cohort revealed that any substance use increased the overall risk of developing schizophrenia (HR 6.04, CI 5.84–6.26). Furthermore, their data showed that the risk was significantly heightened 10–15 years following the initial diagnosis of the SUD (102).

However, similar to the comorbidities discussed earlier in this article, the question of whether a mental precondition causes the development of a SUD or whether the mental illness is a consequence of substance use cannot be readily answered for psychotic disorders. The complexity of this question is probably best illustrated by the scientific discourse on schizophrenia and cannabis (93). For example, although there is a strong relationship between frequency of cannabis use, THC potency, and first-episode psychosis (94), various bio-psycho-social factors appear to influence the development of cannabis use disorder and the onset of psychotic symptoms (93). While cannabis use in adolescence is considered a well-established risk factor for psychosis (94, 103, 104), other biological and environmental factors contribute to the potential manifestation of schizophrenia (105). For example, cannabis use and traumatic childhood experiences seem to synergistically interact to increase the risk of psychotic symptoms in adulthood (106). In addition, a growing body of evidence suggests that genetic factors favor the development of schizophrenia and co-occurring substance use disorders (93). Another finding supports the hypothesis that an increased genetic risk for schizophrenia predicts cannabis initiation (107).

### Overdose and Suicidality

The correlation between adolescent suicide and mental disorders is well-established. Affective disorders, SUD, and past suicide attempts are strongly linked with completed youth suicides (108). Mental disorders and substance use are associated with higher rates of suicidal ideation, suicide attempts and completed suicides (109). Suicidality, “passive” death wishes, “weariness of life”, or “it doesn't matter”-attitudes can become mixed and therefore difficult to disentangle and pose a challenge for parents, caregivers, and clinicians. Hence, the distinction between suicidality and unintentional overdoses, where there is a risk of fatal overdose, is of major clinical importance when working with adolescents or TAY and SUDs. A review of illicit drug use in metropolitan and nonmetropolitan areas and overdose deaths in the United States found an overall decline in illicit drug use, whereas an increase in reports of drug overdose deaths, particularly in rural areas, has been noted (110). In treatment-seeking youth for SUD, high rates of overdose events have been reported, and overdose events were associated with more complex SUD and higher psychiatric comorbidity (111). Yule et al. further found that the likelihood of an overdose event was higher in TAY with two or more SUDs when compared to TAY with only one SUD. Compared to patients without a history of overdose, those with overdose history were more likely to be female and had higher lifetime prevalence of different psychoactive substance use and psychiatric comorbidities (111). Another US analysis of suicide examinations confirmed drug and alcohol use as a suicide risk factor, particularly in Hispanic youth (112). In addition, episodes of heavy drinking and elevated frequency drunkenness are associated with an increased risk of suicide attempts among students (113, 114).

## Discussion

### Findings, Limitations, and Future Directions

Personality disorders, ADHD, and affective disorders appear to be the most common comorbidities in substance use disorders in TAY. In addition, mental disorder comorbidities were common among adolescents and young adults with SUDs and were the rule rather than the exception. From our perspective, the literature confirms a bidirectional, multifactorial relationship. On the one hand, mental disorders promote the development of substance use disorders, and on the other hand, substance use disorders negatively influence mental health problems. In addition, it was found that the literature was very heterogeneous. This shows the complexity of the topic on the one hand. On the other hand, it demonstrates the differences in research approaches as well as how cultural, geographic, and socioeconomic circumstances seem to affect substance use and handling of substances.

A primary strength of this review is the broad perspective that illustrates this topic's complexity. The main limitation of this review is the unsystematic data collection and the narrative style of this article. Therapeutic recommendations were not included in the review, which represents another limitation. However, there are still no generally applicable treatment recommendations. A major challenge will be to provide recommendations for treatment with stimulants in young patients with ADHD and with comorbid addictive disorders or at high risk for developing an addictive disorder as data across the screened literature is highly heterogeneous. Further research is needed to distinguish between patients who benefit from stimulant therapy and those for whom stimulant treatment increases the likelihood of developing problematic substance use or worsen the course of preexisting SUDs. Concerning sedative, hypnotic or anxiolytic use disorders, literature regarding youth is scarce. In fact, in our literature search we did not find appropriate literature for this review. In our view, more research, and the development of therapeutic guidelines for sedatives, hypnotic or anxiolytic use disorders are urgently needed. The development and validation of screening instruments to assess suicide risk in adolescents with substance use disorders represents another necessary task for the future.

## Conclusions

As shown in this review, mental disorders and SUD in TAY are strongly linked. Both SUD and mental illnesses are considered risk factors for drug overdoses and suicides. From our perspective, early identification and intervention for young people with developing or advanced substance use disorders is an essential task for the healthcare system. Early detection and possible interventions of substance use and mental health issues should be easily accessible. Regarding clinical experience of this study group with TAY and SUDs, interdisciplinary cooperation between child and adolescent specialists and adult psychiatry/psychology seems of utmost importance. Furthermore, social workers, general practitioners, school personnel and parents should be integrated when necessary. Raising awareness further and creating appropriate services for a complex and growing target population should be future goals of practitioners and investigators. Efforts should also be made to develop screening and treatment guidelines for TAY with SUDs.

## Author Contributions

PK, MM, and JE did the literature review and wrote the draft. KD, MV, and MW commented on the first draft. PK, MV, and MW commented on the second draft. All authors commented on the final manuscript, which was completed by PK, MM, JE, KD, MV, and MW.

## Conflict of Interest

The authors declare that the research was conducted in the absence of any commercial or financial relationships that could be construed as a potential conflict of interest.

## Publisher's Note

All claims expressed in this article are solely those of the authors and do not necessarily represent those of their affiliated organizations, or those of the publisher, the editors and the reviewers. Any product that may be evaluated in this article, or claim that may be made by its manufacturer, is not guaranteed or endorsed by the publisher.
